# Increased Salience Network Activity in Patients With Insomnia Complaints in Major Depressive Disorder

**DOI:** 10.3389/fpsyt.2018.00093

**Published:** 2018-03-20

**Authors:** Chun-Hong Liu, Jing Guo, Shun-Li Lu, Li-Rong Tang, Jin Fan, Chuan-Yue Wang, Lihong Wang, Qing-Quan Liu, Cun-Zhi Liu

**Affiliations:** ^1^Beijing Hospital of Traditional Chinese Medicine, Beijing Institute of Traditional Chinese Medicine, Capital Medical University, Beijing, China; ^2^Beijing Key Laboratory of Mental Disorders, Department of Radiology, Beijing Anding Hospital, Capital Medical University, Beijing, China; ^3^Acupuncture and Moxibustion Department, Beijing Hospital of Traditional Chinese Medicine, Capital Medical University, Beijing, China; ^4^Department of Psychiatry, Icahn School of Medicine at Mount Sinai, New York, NY, United States; ^5^Department of Psychiatry, University of Connecticut Health Center, Farmington, CT, United States

**Keywords:** insomnia, depression, resting-state, low-frequency fluctuation, salience networks

## Abstract

**Background:**

Insomnia is one of the main symptom correlates of major depressive disorder (MDD), but the neural mechanisms underlying the multifaceted interplay between insomnia and depression are not fully understood.

**Materials and methods:**

Patients with MDD and high insomnia (MDD-HI, *n* = 24), patients with MDD and low insomnia (MDD-LI, *n* = 37), and healthy controls (HCs, *n* = 51) were recruited to participate in the present study. The amplitude of low-frequency fluctuations (ALFF) during the resting state were compared among the three groups.

**Results:**

We observed ALFF differences between the three groups in the right inferior frontal gyrus/anterior insula (IFG/AI), right middle temporal gyrus, left calcarine, and bilateral dorsolateral prefrontal cortex (dlPFC). Further region of interest (ROI) comparisons showed that the increases in the right IFG/AI reflected an abnormality specific to insomnia in MDD, while increases in the bilateral dlPFC reflected an abnormality specific to MDD generally. Increased ALFF in the right IFG/AI was also found to be correlated with sleep disturbance scores when regressing out the influence of the severity of anxiety and depression.

**Conclusion:**

Our findings suggest that increased resting state ALLF in IFG/AI may be specifically related to hyperarousal state of insomnia in patients with MDD, independently of the effects of anxiety and depression.

## Introduction

Major depressive disorder (MDD) is characterized by a sustained depressive mood, anhedonia, and sleep abnormalities, alongside a number of motivational and social behaviors (1). Globally, it is a major cause of disability (2). Epidemiological studies have shown that MDD frequently co-occurs with insomnia and insomnia can persist into the remission or recovery stage (3–6). Importantly, the relationship between insomnia and depression is bidirectional with the severity of sleep disturbances positively correlated to the overall severity of depression and to a poor quality of life (7). Insomnia may also increase the severity of depression and the risk of suicidality (8). Indeed, insomnia, which is a disorder that is independent of depression (9), has a prognostic value as a risk factor for subsequent depressive episodes (3, 10). In longitudinal studies of adults with MDD, insomnia has been shown to increase the risk of recurrence of new depressive episodes twofold to fourfold (11, 12). Additionally, the re-emergence of insomnia can predict the recurrence of a new depressive episode (13). Insomnia can also lead to poor responses to various forms of treatment for depression (3). Treatments for insomnia might help with treatments of depressive symptoms (14). Likewise, insomnia significantly affects the symptoms that are correlated with MDD (15). So, as there appears to be an interplay between insomnia and depression that is multifaceted, examining the specific underlying brain abnormalities associated with insomnia in patients with MDD, could produce data that may lead to the availability of individualized therapies for patients with MDD. However, the neural mechanisms underlying insomnia and MDD comorbidity are currently unclear.

Intrinsic functional connectivity (FC) studies show that several large-scale brain networks have been considered as potential neural substrates in MDD, including the default mode network, the frontoparietal and dorsal attention network, and the salience networks [for a review, see Ref. (16, 17)]. The majority of the above studies have focused on the relationship between three core intrinsic connectivity networks, suggesting that psychiatric disorders, including MDD, could be explained in part by the triple network model (18). However, there is as yet no systematic evidence for the specific abnormalities that may be associated with MDD where symptoms of insomnia coexist. Within the triple network, the salience network, which mainly involves the amygdala, the anterior insula (AI) and the dorsal anterior cingulate cortex (dACC) has recently been identified as playing an essential role in both insomnia and MDD (18, 19). The right AI within the salience network is predominantly responsible for the integration of autonomic, visceromotor, emotional and interoceptive responses (18). As a result, the salience network is postulated to mediate the emotional, vegetative and somatic aspects of depression, including sleep disturbances (20). Using simultaneous resting-state functional magnetic resonance imaging (rsfMRI) and electroencephalogram (EEG) recordings, Chen et al. (21) observed that patients with insomnia who were not depressed had increased ventral insula co-activation within the salience system when they were compared with healthy controls (HCs) at rest. Interestingly, the subjects with insomnia in Chen et al.’s study (21) had higher anxiety and depression scores than the HC group, although the scores were below clinical thresholds. The increased co-activation of the insula/salience network observed in patients with insomnia could be indicative of unconscious anxiety/depression, or of insufficient gating of sensory stimuli (22). Using an interoceptive attention task, Avery et al. (23) demonstrated a decrease in the insular activation in MDD patients, but the anxiety level in this particular MDD group was not examined. Interestingly, one of our previous studies on depressed patients with anxiety also revealed increased resting-state activity in the AI (close to the middle insula region) (24). However, Guo et al. found that there was a decrease in the short-range strength of FC in the right insula in drug-naïve MDD patients and no regional activity was observed in the insula (25, 26). So, whether increased co-activity of the AI within the salience system is related to anxiety, depression, or insomnia remains unclear; it was anticipated that analyzing regional spontaneous brain activity in MDD patients with insomnia should assist in clarifying this.

A number of techniques have been developed for the analysis of the data generated by rsfMRI, including FC, regional homogeneity (ReHo), the amplitude of low-frequency fluctuations (ALFF), and the fractional amplitude of low-frequency fluctuations (fALFF). ReHo depicts the local coherency of a given voxel to those of neighboring voxels and is limited in its usefulness between spatially distant brain domains (27). Traditional seed-based FC was initially used to measure correlations based on low-frequency fluctuating signals (28). ALFF measures the absolute strength or intensity of low-frequency oscillations and has a higher test–retest reliability in gray matter than white matter (29). ALFF has repeatedly been reported to reflect concurrent local neuronal activity (24, 30), and is an effective technique for examining the fluctuations in disease-related regional spontaneous activity strength during the resting state. It has been demonstrated to be abnormal in a number of psychiatric disorders, including MDD and bipolar disorder (27, 31).

To validate the above hypothesis, it is essential to assess whether the regional spontaneous brain activity in the salience network prevails when a comorbidity of insomnia and depression exists. Interestingly, a reduction in γ-aminobutyric acid in dACC within the salience network has been identified in both primary insomnia and MDD using single-voxel proton magnetic spectroscopy (1H-MRS) (32). As strong links between insomnia and MDD have already been established, we hypothesized that patients with comorbid insomnia and MDD would display abnormal ALFF in regional spontaneous activity, especially in the salience network. Confirmation of this will provide important information for furthering understanding of the mechanisms underlying MDD and high insomnia (MDD-HI). As individuals with MDD-HI may not necessarily be in a highly aroused or ruminative state, but would be expected to be lethargic or mindless, we hypothesized that increased right insular activity would be observed during the resting state in MDD-HI patients. We hypothesized further that these alterations may be an essential biomarker for insomnia with MDD after adjusting for anxiety and depression as a covariate. The aim of the current study was to clarify these issues and so enhance understanding of the neural mechanisms in the high-insomnia subtype of MDD.

## Materials and Methods

### Participants

The present study was approved by the Research Ethics Review Board of Beijing Anding Hospital, Capital Medical University and State Key Laboratory of Cognitive Neuroscience and Learning, Beijing Normal University. After study procedures were fully explained to participants, they gave written consent before experiments were initiated.

Subsets of the data used here have been used in previous studies (24). The participants included 24 MDD-HI and 37 patients with MDD and low insomnia (MDD-LI) who were outpatients and inpatients at Beijing Anding Hospital, Capital Medical University. Also, 51 age-, gender-, education-matched, and right-handed HCs were included in the present study. All 61 participants with MDD were diagnosed by two experienced psychiatrists using the Structured Clinical Interview (SCID) for the Diagnostic and Statistical Manual for Mental Disorders, Fourth Edition (DSM-IV) (33). Sleep disturbances (consisting of inability to fall asleep, night waking, and waking too early) were evaluated using the 17-item Hamilton Depression Rating Scale (HAMD) (34, 35) and the sleep disturbance factor of the HAMD insomnia subscale, including items 4 (insomnia-early), 5 (insomnia-middle), and 6 (insomnia-late) (36–38). According to Park et al. (37), the cutoff point for MDD patients with “low insomnia” was an insomnia level ≤3 on the HAMD subscale and for “high insomnia” was an insomnia level ≥4. The baseline depressive symptoms were derived from the adjusted HAMD scores in which the scores for the sleep items (questions 4–6) were removed to measure severity of depression and to minimize any effects of sleep disturbances on severity of depression (37–40). The severity of anxiety was evaluated based on the Hamilton Anxiety Rating Scale (HAMA) (34). Participants’ details are presented in Table 1. Inclusion criteria have been previously reported (31), with all participants: (1) 18–60 years old; (2) right handed; (3) meeting the DSM-IV diagnostic criteria for MDD; (4) no history of current serious medical or neurological illness; (5) no history of other psychiatric disorders (e.g., schizophrenia and obsessive-compulsive disorder) or an anxiety disorder (e.g., panic disorder, generalized anxiety disorder or a specific phobia); (6) no history of trauma resulting in loss of consciousness; (7) no diagnosis of dementia or developmental disorder; and (8) no history of alcohol and substance abuse or dependence. The HCs were interviewed with the non-patient edition of SCID. Exclusion criteria included the presence of any DSM-IV axis-I diagnosis, any current serious medical or neurological illness, a history of neurological or neuropsychiatric illness, a history of head trauma with loss of consciousness, and a positive history of a major psychiatric disorder, dementia, or mental retardation.

**Table 1:** Group demographics and clinical measures.

Measure (mean ± SD)	MDD-LI (*n* = 37)	MDD-HI (*n* = 24)	HC (*n* = 51)	Statistical value	*p*-Value
Age, years	34.27 ± 11.23	41.33 ± 12.51	35.53 ± 12.53	2.69	0.07^#^
Years of education	15.11 ± 2.99	14.17 ± 2.93	15.76 ± 2.36	2.88	0.06^#^
Gender (male/female)	9/28	12/12	23/28	5.35	0.07Δ
Illness duration (years)	5.58 ± 6.21	9.94 ± 11.15		1.96	0.06*
Number of depressive episodes	2.24 ± 1.34	2.46 ± 2.06		0.49	0.62*
HAMD	13.57 ± 7.00	26.17 ± 6.93		6.89	<0.001^#^
Adjusted HAMD	9.24 ± 6.21	16.08 ± 5.93		4.28	<0.001^#^
HAMA	11.70 ± 7.75	20.04 ± 8.85		3.88	<0.001^#^
Sleep disturbance	2.19 ± 0.74	5.04 ± 0.91		13.45	<0.001^#^
Antidepressants	42	19			
SSRI	26	12			
SNRI	7	5			
Mirtazapine	1	0			
Trazodone	1	2			
TCA	3	0			
Flupentixol and melitracen tetracyclic	4	1			
Antipsychotics	10	2			
Quetiapine	6	2			
Resperidone	2	0			
Aripiprazole	2	0			
Benzodiazepines	3	3			
Lonazepan	1	3			
Oxazepam	2	0			
Medication-free	0	4			

*Adjusted Hamilton Depression Rating Scale (HAMD) score means HAMD scores after omission of sleep questions. MDD-HI, MDD patients with high insomnia; MDD-LI, MDD patients with low insomnia; HC, healthy controls; HAMD, Hamilton Depression Rating Scale; HAMA, Hamilton Anxiety Rating Scale; SSRI, selective serotonin reuptake inhibitor; SNRI, selective serotonin and noradrenalin reuptake inhibitor; TCA, tricyclic antidepressant*.

^#^p-Values for one-way ANOVA, *p-values for two-sample t-tests, and ^Δ^p-values for chi-square test.

### Image Acquisition

Images were acquired using a Siemens Trio 3-Tesla MRI scanner at the National Key Laboratory for Cognitive Neuroscience and Learning, Beijing Normal University, Beijing. All rsfMRI data were acquired using an echo-planar imaging (EPI) sequence with the following parameters: 33 axial slices, repetition time (TR) = 2,000 ms, echo time (TE) = 30 ms, flip angle (FA) = 90°, thickness/gap = 3.5/0.6 mm, field of view (FOV) = 220 mm^2^ × 220 mm, and matrix size = 64 mm × 64 mm with 240 volumes. A resting state was defined as when subjects performed no specific cognitive tasks during scanning. All participants were instructed to be still, shut their eyes, clear their minds and not to fall asleep.

### Image PreProcessing

Image preprocessing was performed using Data Processing Assistant and Resting-State fMRI (DPARSF) Advanced Edition^1^ (41) and the Statistical Parametric Mapping software package (SPM8)^2^ MATLAB (MathWorks) toolboxes. The first 10 volumes of each participant’s functional time points were removed for signal stabilization to allow them to adapt to scanner noise. Slice timing and head motion correction were conducted first. Head motion was evaluated by the realigning parameters that were estimated by SPM and reported in “ExcludeSubjects.txt” in the “RealignParameter” directory. In addition the mean frame-wise displacement was calculated to measure the scrubbing-related microhead motion of each subject. The largest mean frame-wise displacement (FD, Jenkinson) of all the subjects was <0.2 mm (42). No participant was excluded from additional analysis by excessive motion criterion (>2 mm of translation or >2° of rotation in any direction) and frame-wise displacement values. Then, the structural image of each participant was coregistered to the head motion-corrected EPI image. The coregistered structural images were segmented using a unified segmentation algorithm, which significantly improved the accuracy of spatial normalization, and were then transformed into standard Montreal Neurological Institute (MNI) space. The EPI images were also spatially normalized to MNI space by applying the parameters of structural image normalization and were resampled to a voxel size of 3 mm × 3 mm × 3 mm. Finally, EPI images were spatially smoothed with a Gaussian kernel of 4-mm full width at half maximum (FWHM). In addition, linear trend removal and temporal band-pass filtering (0.01–0.08 Hz) were performed. At last, nuisance covariates, e.g., head motion parameters, global mean time courses, white matter time courses, and cerebrospinal fluid time courses were regressed out.

### ALFF Map Calculation

The filtered time series of each voxel was transformed into the frequency domain using a fast Fourier transformation, and the power spectrum was obtained. The square root was calculated at each frequency of the power spectrum because the power of a given frequency is proportional to the square of the amplitude of this frequency. The averaged square root across 0.01–0.08 Hz at each voxel was taken as the ALFF. The ALFF value of each voxel was also divided by the raw mean ALFF value for standardization purposes in order to reduce the effects of variability across participants (43). The mean individual ALFF maps were analyzed statistically.

### Statistical Analyses

The most updated bug-fixed version of the Resting State functional magnetic resonance imaging (fMRI) Data Analysis Toolkit AlphaSim program in AFNI for multiple comparisons (RESTplus 1.1_20160113) was used for statistical analyses. We performed one-way analysis of variance (ANOVA) across the ALFF among the three groups with sex, age, education level, adjusted HAMD, and HAMA scores as covariates. The statistically corrected threshold of *p* < 0.05 within the whole-brain mask (size, 276,133 mm^3^) was determined with Monte Carlo simulations [parameters: single voxel *p* = 0.01, FWHMx = 4.575 mm, FWHMy = 4.564 mm, FWHMz = 4.508 mm, cluster size = 486 mm^3^ and 1,000 iterations (44)]. For the regions showing significant differences among the three groups, we conducted further region of interest (ROI) analysis across the MDD-HI, MDD-LI and HC groups to investigate whether these regions showed abnormalities specific to insomnia or MDD. Additionally, voxel-wise Pearson’s correlation analyses were performed to indicate the relationships between the ALFFs with sleep disturbance scores with age, gender, educational level, HAMA, and adjusted HAMD scores as covariates in pooled MDD patients (including both the MDD-HI and MDD-LI groups).

## Results

### Demographic and Clinical Characteristics

In Table 1, the demographic details and clinical characteristics of the participants in the study are summarized. The individuals in the MDD-HI, MDD-LI, and HC groups were well matched for age, sex distribution and years in education. The adjusted HAMD, sleep disturbance, and HAMA scores were highly correlated (*r* = 0.803 and *p* < 0.001 for adjusted HAMD and sleep disturbance scores; *r* = 0.842 and *p* < 0.001 for adjusted HAMD and HAMA scores, and *r* = 0.567 and *p* < 0.001 for HAMA and sleep disturbance scores) in pooled MDD patients. Mean FD did not differ among individuals in the MDD-HI (0.080 ± 0.032), MDD-LI (0.077 ± 0.033), and HC (0.077 ± 0.033) groups in the final sample (*F*[2, 110] = 0.328, *p* = 0.744).

### Differences in ALFF Values Between Groups

**Figure 1:**
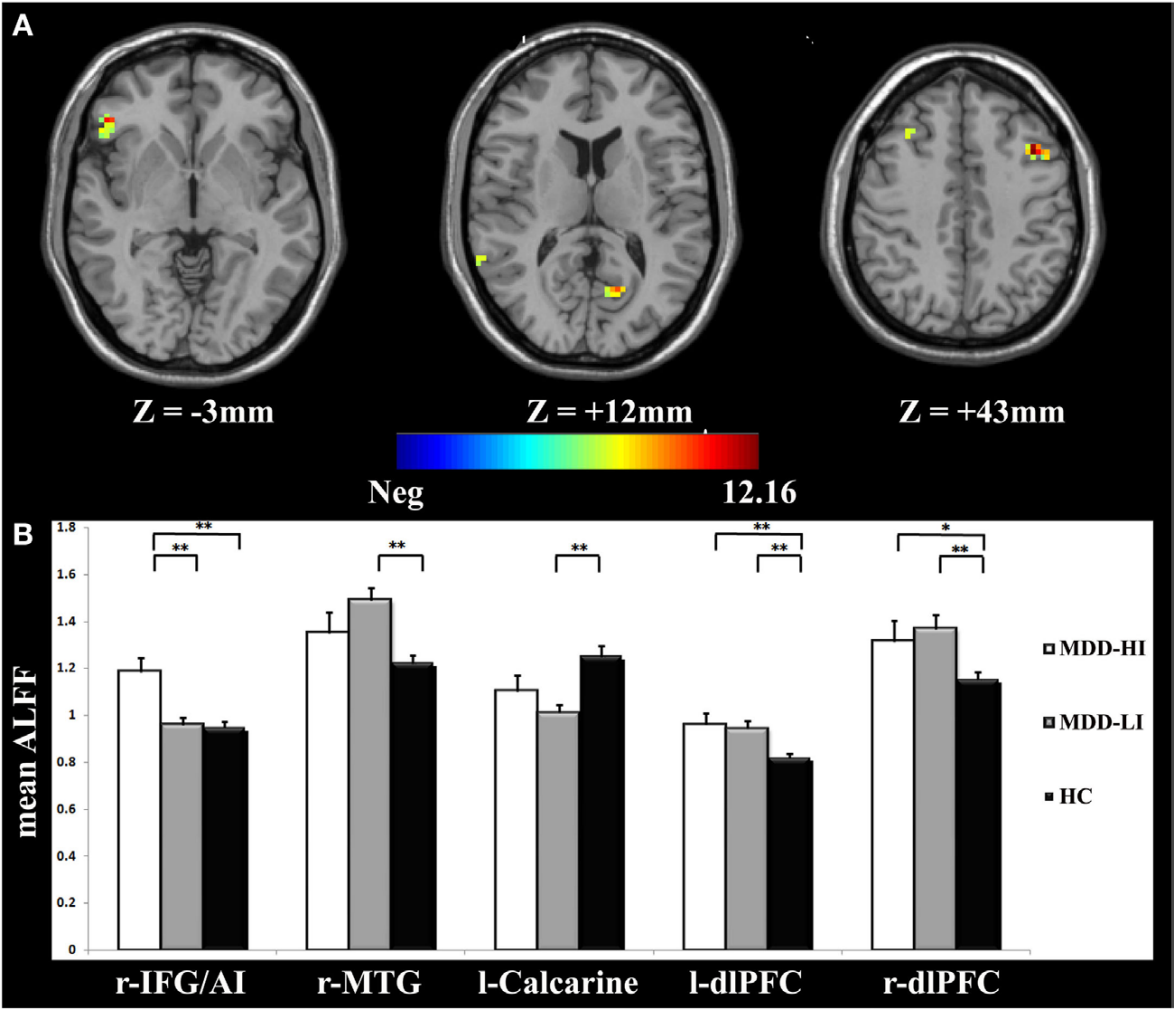
**(A)** Analysis of variance (ANOVA) of the amplitude of low-frequency fluctuation (ALFF) values of the three groups with the covariates of age, gender, educational level, and adjusted Hamilton Depression Rating Scale (HAMD) scores. The color bar represents the *F*-value from ANOVA. The numbers below the images refer to the *z*-coordinates according to the Montreal Neurological Institute (MNI) atlas. The statistical threshold was set at |*F*| = 4.83 (*p* = 0.01) with a cluster size of 486 mm^3^, which corresponded to an AlphaSim corrected *p* < 0.05. **(B)** The bar graphs represent mean ALFF values in each region of interest (ROI) for r-IFG/AI, r-MTG, l-Calcarine, l-dlPFC, and r-dlPFC, respectively. r-IFG/AI, right inferior frontal gyrus/anterior insula; r-MTG, right middle temporal gyrus; l-Calcarine, left calcarine; l- dlPFC, left dorsolateral prefrontal cortex; r-dlPFC, right dorsolateral prefrontal cortex.

One-way ANOVA demonstrated that there were significant differences among the three groups (*p* < 0.05, corrected) in the right inferior frontal gyrus/anterior insula (IFG/AI), the right middle temporal gyrus, the left calcarine, and the bilateral dorsolateral prefrontal cortex (dlPFC) (Figure 1A; Table 2).

**Table 2:** Brain areas with significant differences in the ALFF values among three groups.

Brain regions	Side	Brodmann areas	Talairach coordinates			*K*	*F*-value
			*x*	*y*	*z*		
IFG/AI	R	47	51	33	−3	29	10.08
Middle temporal gyrus	R	22	63	−51	9	36	8.28
Calcarine	L		−15	−66	12	18	9.05
dlPFC	R		21	30	33	26	9.03
dlPFC	L		−39	15	42	22	12.16

*ALFF, amplitude of low-frequency fluctuation; K, cluster number; IFG, inferior frontal gyrus; AI, anterior insula; dlPFC, dorsolateral prefrontal cortex*.

### ROI-Wise ALFF Comparisons

We conducted ROI analyses within the ALFF differences among the three groups. The ALFF values of the five brain regions with significant difference in Section “Differences in ALFF Values Between Groups” were averaged, yielding an ROI-wise mean ALFF value. The MDD-HI group had significantly increased ALFF values in the right IFG/AI region (*p* < 0.001) when they were compared with the ALFF values in the MDD-LI group. The MDD-HI group had significantly increased ALFF values in the right IFG/AI (*p* < 0.001), the left dlPFC (*p* < 0.001), and the right dlPFC (*p* < 0.041) when compared with the HC group. In contrast, the MDD-LI group exhibited significantly increased ALFF values in the right middle temporal gyrus (*p* < 0.001) and the bilateral dlPFC (*p* < 0.001), as well as decreased ALFF values in the left calcarine (*p* < 0.001) relative to the HC group. These results are illustrated in Figure 1B. More significantly, it was determined that the right IFG/AI displayed alterations that were more specific to insomnia in MDD and increased ALFF values in the bilateral dlPFC were related more in general to MDD. The mean Cohen’s *d* in the right IFG/AI for MDD-HI vs. MDD-LI groups, MDD-HI vs. HC groups and MDD-LI vs. HC groups were 1.12, 1.05, and −0.03, respectively. The effect–size correlation, *ry*, in the right IFG/AI for MDD-HI vs. MDD-LI groups, MDD-HI vs. HC groups, and MDD-LI vs. HC groups were 1.12, 0.47, and −0.02, respectively.

### Correlation Analyses

**Figure 2:**
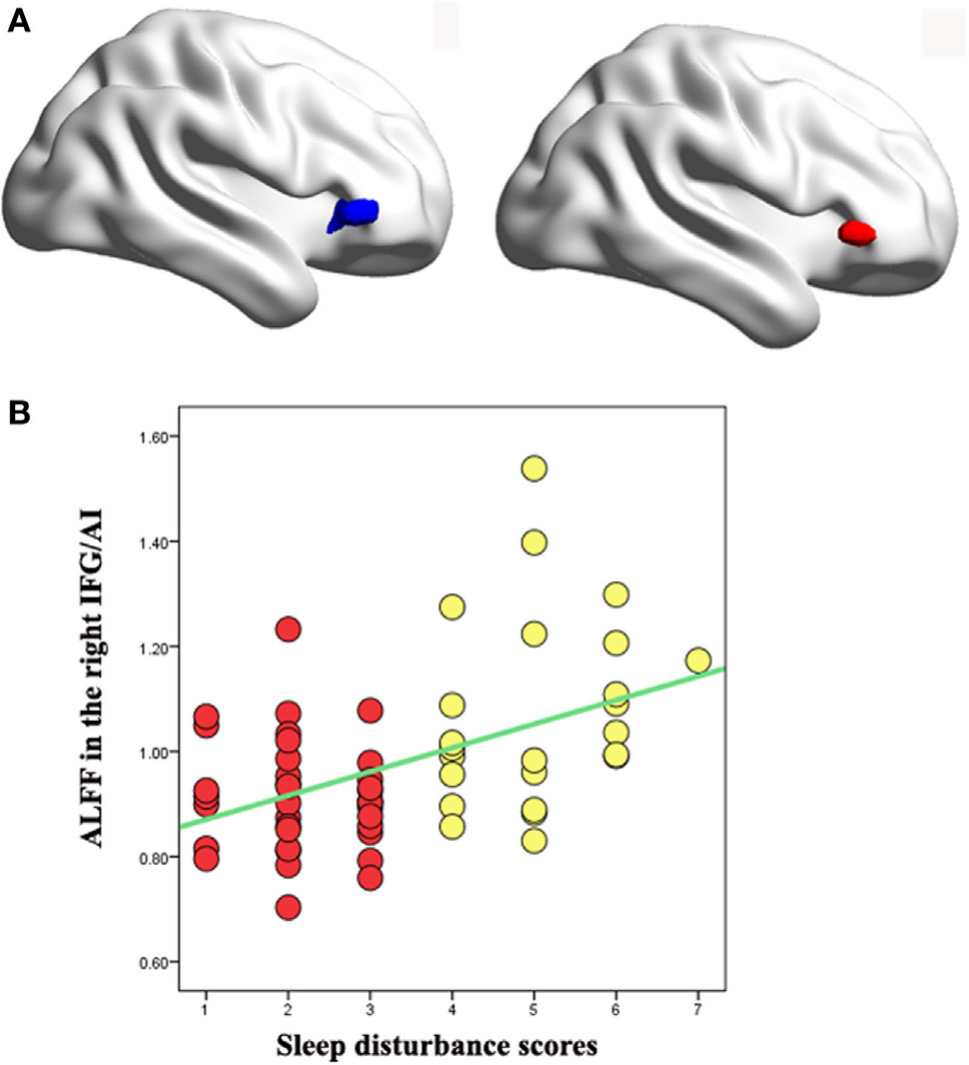
**(A)** Left: voxel-wise correlation analysis between the amplitude of low-frequency fluctuation (ALFF) values and sleep disturbance scores revealed positive correlation in the right inferior frontal gyrus/anterior insula (IFG/AI) in pooled major depressive disorder (MDD) patients with high and low insomnia (peak coordinate: 48, 33, 0) with age, gender, educational level, anxiety, and adjusted depression scores as covariates (navy blue). Right: the right IFG/AI region from analysis of variance (ANOVA) of the three groups with the covariates of age, gender, educational level, and adjusted depression scores (red). The two right IFG/AI clusters obviously overlapped. **(B)** Region of interest (ROI) analysis of the correlation analysis between the right IFG/AI ALFF values and the sleep disturbance scores confirmed that the results from the whole-brain analysis were not driven by outliers. Yellow dots represent MDD patients with high insomnia, and red dots represent MDD patients with low insomnia.

Voxel-wise regression analyses indicated that the increased ALFF values in the right IFG/AI (peak coordinate: 48, 33, 0) and in the right dACC (peak coordinate: 12, 30, 33) were significantly correlated with the sleep disturbance scores of pooled MDD patients (after controlling for the anxiety and adjusted depression scores; Figure 2A). ROI analysis confirmed that this correlation was not due to outliers (Figure 2B). The detailed correlation results between the ALFF measurements and the severity of sleep disturbance scores at the whole-brain level are presented in Table 3.

**Table 3:** Voxel-wise correlation analysis between the ALFF values and the sleep disturbance scores in pooled MDD patients including both low and high insomnia.

Brain region		Side	Talairach coordinates			Voxels	*r*	*p*
			*x*	*y*	*z*			
**ALFF**	**Positive correlation**							
	IFG/AI	Right	48	33	0	47	0.49	<0.01
	Middle cingulum	Right	12	30	33	18	0.54	<0.01

*ALFF, amplitude of low-frequency fluctuation; IFG/AI, inferior frontal gyrus/anterior insula*.

## Discussion

In the present study, the alterations in resting-state activity specific to insomnia in MDD were examined by comparing the changes in ALFF among subjects with MDD-HI, MDD-LI, and HC. MDD-HI patients had significantly increased ALFF values in the right IFG/AI when compared with MDD-LI patients. Furthermore, increased IFG/AI activity was correlated with the severity of insomnia symptoms after controlling for anxiety and adjusted depression scores. More importantly, it was found that the right IFG/AI is the key node of the salience network. Taken together, the results presented here suggest that abnormalities in the salience network are profoundly involved in the sleep disturbances associated with MDD. In addition, increased ALFF values in the bilateral dlPFC were consistently demonstrated to exist in both the MDD-HI and MDD-LI groups when compared with the HC group. This suggests that increased ALFF values in the dlPFC represent the characteristic physiological change associated with MDD.

The salient network is thought to be responsible for the dynamic switching between the default mode network and the interactions of the central executive network (45), hence, the central role and relevance of the salience network in MDD has been previously described (46, 47). The salience network can be further subdivided into the dorsal and ventral salience networks (48, 49). The dorsal salience network is related to attention and the switching between cognitive resources, while the ventral system is related to emotional processing (48, 49). Likewise, the insula has been parcellated into the dorsal AI, ventral AI and posterior insula by Deen et al. (50), as in a report by Cauda et al. (51). The dorsoanterior insula is associated with cognitive processes including monitoring the interoceptive state and the ventroanterior insula is related to emotional processing (52). In the present study, MDD-HI patients had significantly increased ALFF values in the right IFG/AI relative to MDD-LI patients. The increased IFG/AI and dACC activity was correlated with the severity of the symptoms of insomnia after controlling for anxiety and the adjusted depression scores. The increased dorso-AI activity and its correlation with the severity of insomnia might indicate that the salience network may be specifically related to hyperarousal state of insomnia in patients with MDD, independently of the effects of anxiety and depression.

When combining the findings for the MDD-HI group and the MDD-LI group and comparing them to the HC group, significantly increased ALFF values in the right middle temporal gyrus and bilateral dlPFC were noted, but decreased ALFF values in the left calcarine. These results are largely consistent with the findings of Liu et al. (53), where ALFF values were investigated in 30 treatment-naïve MDD subjects. It was found that MDD patients had significantly increased ALFF values in the bilateral ventral/dorsal ACC, the left dlPFC, the left superior frontal cortex, and the left inferior parietal cortex, as well as decreased ALFF values in the bilateral occipital cortex, the cerebellum and the right superior temporal cortex. The regions affected are largely located in the cortico-limbic circuits, as demonstrated by previous authors (18, 54). Interestingly, analysis of ROI demonstrated that an increased right dlPFC ALFF value is a consistent finding across MDD groups with HI and LI. The involvement of the dlPFC in major depression has been a primary focus of previous studies. Our research group and others have demonstrated that subjects with MDD have attenuated activation or low metabolism in the dlPFC while performing cognitive tasks (55, 56). The data on the dlPFC reported here are in accordance with the general finding that dlPFC activity may be related to a depressive state and might, therefore, serve as a neuroimaging marker of MDD (57).

The HAMD sleep items evaluate three sleep periods, while the Pittsburgh Insomnia Rating Scale evaluates day time sleepiness/dysfunction. The HAMD sleep items may simplify insomnia and place less emphasis on patients with only a single dimension of insomnia, which may be the case in a quarter of MDD patients (58). Moreover, HAMD sleep items are widely recognized to be well correlated with sleep diaries (36). The Pittsburgh Insomnia Rating Scale is a more subjective rating of sleep quality including insomnia, which takes into account subjective ratings of sleep, sleep timings, and sleep duration, as well as daytime dysfunction (38). Our definition of insomnia using the three sleep questions in the HAMD has also been adopted as the main objective measure of insomnia in MDD (36). So, the definition of insomnia based on questions from the HAMD subscales can be considered to be the main objective measure of insomnia in MDD.

The present study has several limitations. First, almost none of the patients were medication-free at the time of the scans due to serious practical and ethical issues, so possible confounding effects of medication could be not ruled out. Also, as detailed lifetime data were not collected (e.g., duration of medication and dose), we could not rule out the potential impact of medication by including it as a covariate in the analyses. Given the fact that the current findings concerning depression are consistent with previous work in treatment-naïve MDD patients (59), it could be argued that the major findings of this study are valid, regardless of the medication issue. Clearly, further studies in treatment-naïve patients with and without symptoms of insomnia are necessary to confirm the current findings. A second limitation of the present study is that it did not include insomnia patients not suffering with MDD. To best clarify the underlying neural mechanisms specifically related to MDD with insomnia and to MDD without insomnia, future studies should involve a larger number of MDD patients as well as insomniacs without MDD. Third, follow-up of MDD patients is necessary to determine which are likely to develop HI. Fourth, the mean ages of the MDD-LI, MDD-HI and HC groups were 34.27 (SD = 14.6), 41.33 (SD = 12.51), and 35.53 (SD = 12.53), respectively. Although the mean age of the MDD-HI group was above that of the MDD-LI group, they were both representative of the adult population with MDD (60). Fifthly, we performed family wise error (FWE) corrected using threshold-free cluster enhancement (TFCE) in FSL (61) and multiple comparisons using false discovery rate (FDR) correction. However, the results were negative. The final limitation of the present study is that insomnia was only measured using the insomnia factor in the HAMD. Further investigations should now be conducted employing additional tools to assess insomnia and sleep, such as the Pittsburgh Insomnia Rating Scale, polysomnography, or sleep diaries to improve the accuracy of the insomnia measurements.

In summary, the key findings of the present study were increased intrinsic neural oscillation within the right IFG/AI in MDD patients with HI, which was independent of symptoms of anxiety or depression. Although we cannot rule out the influence of medication on intrinsic neural oscillations, consistent findings in the IFG/AI between the present study and previous reports in the literature suggest that there is a high possibility of the involvement of the salience network in insomnia, rather than a medication effect. Confirmation of the findings presented here will help to establish whether there is an insomnia subtype of MDD patient. The present results also extend those of previous studies and demonstrate that increased intrinsic neural oscillations in the right dlPFC during the resting state is a characteristic change in the depressive state that merits further investigation as a potential imaging marker for MDD.

## Ethics Statement

The present study was approved by the Research Ethics Review Board of Beijing Anding Hospital, Capital Medical University, and State Key Laboratory of Cognitive Neuroscience and Learning, Beijing Normal University.

## Author Contributions

C-HL and JG designed the study, along with S-LL, L-RT, JF, C-YW, LW, and Q-QL. C-HL, JG, S-LL, L- T, and C-YW collected the original imaging data. C-HL, JF, and LW managed and analyzed the imaging data. C-HL, L-RT, JF, C-YW, LW, and Q-QL wrote the manuscript. All authors contributed to and have approved the final manuscript.

## Conflict of Interest Statement

There are no conflicts of interest, financial or otherwise, related directly or indirectly to this work.

## Footnotes

^1^http://rfmri.org/DPARSF.

^2^http://www.fil.ion.ucl.ac.uk/spm.

## References

[1] Whiteford HA Degenhardt L Rehm J Baxter AJ Ferrari AJ Erskine HE Global burden of disease attributable to mental and substance use disorders: findings from the Global Burden of Disease study 2010. Lancet (2013) 382:1575–786. 10.1016/S0140-6736(13)61611-6 23993280

[2] Paulus MP Stein MB. An insular view of anxiety. Biol Psychiatry (2006) 60:383–7. 10.1016/j.biopsych.2006.03.042 16780813

[3] Baglioni C Battagliese G Feige B Spiegelhalder K Nissen C Voderholzer U Insomnia as a predictor of depression. A meta-analytic evaluation of longitudinal epidemiological studies. J Affect Disord (2011) 135:10–9. 10.1016/j.jad.2011.01.011 21300408

[4] Riemann D Voderholzer U. Primary insomnia: a risk factor to develop depression? J Affect Disord (2003) 76:255–9. 10.1016/S0165-0327(02)00072-1 12943956

[5] Riemann D. Insomnia and comorbid psychiatric disorders. Sleep Med (2007) 8(Suppl 4):S15–20. 10.1016/S1389-9457(08)70004-2 18346672

[6] Tsuno N Besset A Ritchie K Sleep and depression. J Clin Psychiatry (2005) 66:1254–69. 10.4088/JCP.v66n1008 16259539

[7] McCall WV Reboussin BA Cohen W. Subjective measurement of insomnia and quality of life in depressed inpatients. J Sleep Res (2000) 9:43–8. 10.1046/j.1365-2869.2000.00186.x 10733688

[8] Walker MP. The role of sleep in cognition and emotion. Ann N Y Acad Sci (2009) 1156:168–97. 10.1111/j.1749-6632.2009.04416.x 19338508

[9] Harvey AG. Insomnia: symptom or diagnosis? Clin Psychol Rev (2001) 21:1037–59. 10.1016/S0272-7358(00)00083-0 11584515

[10] Jindal RD Thase ME. Treatment of insomnia associated with clinical depression. Sleep Med Rev (2004) 8:19–30. 10.1016/S1087-0792(03)00025-X 15062208

[11] Breslau N Roth T Rosenthal L Andreski P. Sleep disturbance and psychiatric disorders: a longitudinal epidemiological study of young adults. Biol Psychiatry (1996) 39:411–8. 10.1016/0006-3223(95)00188-3 8679786

[12] Chang PP Ford DE Mead LA Cooper-Patrick L Klag MJ. Insomnia in young men and subsequent depression. The Johns Hopkins Precursors study. Am J Epidemiol (1997) 146:105–14. 10.1093/oxfordjournals.aje.a009241 9230772

[13] Manber R Chambers AS. Insomnia and depression: a multifaceted interplay. Curr Psychiatry Rep (2009) 11:437–42. 10.1007/s11920-009-0066-1 19909664

[14] Howland RH. Sleep interventions for the treatment of depression. J Psychosoc Nurs Ment Health Serv (2011) 49:17–20. 10.3928/02793695-20101208-01 21175118

[15] Yao Z Wang L Lu Q Liu H Teng G. Regional homogeneity in depression and its relationship with separate depressive symptom clusters: a resting-state fMRI study. J Affect Disord (2009) 115:430–8. 10.1016/j.jad.2008.10.013 19007997

[16] Kaiser RH Andrews-Hanna JR Wager TD Pizzagalli DA. Large-scale network dysfunction in major depressive disorder: a meta-analysis of resting-state functional connectivity. JAMA Psychiatry (2015) 72:603–11. 10.1001/jamapsychiatry.2015.0071 25785575 PMC4456260

[17] Mulders PC van Eijndhoven PF Schene AH Beckmann CF Tendolkar I. Resting-state functional connectivity in major depressive disorder: a review. Neurosci Biobehav Rev (2015) 56:330–44. 10.1016/j.neubiorev.2015.07.014 26234819

[18] Menon V. Large-scale brain networks and psychopathology: a unifying triple network model. Trends Cogn Sci (2011) 15:483–506. 10.1016/j.tics.2011.08.003 21908230

[19] Spiegelhalder K Regen W Baglioni C Nissen C Riemann D Kyle SD. Neuroimaging insights into insomnia. Curr Neurol Neurosci Rep (2015) 15:9. 10.1007/s11910-015-0527-3 25687698

[20] Groenewold NA Opmeer EM de Jonge P Aleman A Costafreda SG. Emotional valence modulates brain functional abnormalities in depression: evidence from a meta-analysis of fMRI studies. Neurosci Biobehav Rev (2013) 37:152–63. 10.1016/j.neubiorev.2012.11.015 23206667

[21] Chen MC Chang C Glover GH Gotlib IH. Increased insula coactivation with salience networks in insomnia. Biol Psychol (2014) 97:1–8. 10.1016/j.biopsycho.2013.12.016 24412227 PMC3961550

[22] Hairston IS Talbot LS Eidelman P Gruber J Harvey AG. Sensory gating in primary insomnia. Eur J Neurosci (2010) 31:2112–21. 10.1111/j.1460-9568.2010.07237.x 20529120

[23] Avery JA Drevets WC Moseman SE Bodurka J Barcalow JC Simmons WK. Major depressive disorder is associated with abnormal interoceptive activity and functional connectivity in the insula. Biol Psychiatry (2014) 76:258–66. 10.1016/j.biopsych.2013.11.027 24387823 PMC4048794

[24] Liu CH Ma X Song LP Fan J Wang WD Lv XY Abnormal spontaneous neural activity in the anterior insular and anterior cingulate cortices in anxious depression. Behav Brain Res (2015) 281:339–47. 10.1016/j.bbr.2014.11.047 25513974

[25] Guo W Liu F Chen J Wu R Zhang Z Yu M Decreased long-and short-range functional connectivity at rest in drug-naive major depressive disorder. Aust N Z J Psychiatry (2016) 50:763–9. 10.1177/0004867415617835 26607302

[26] Guo W Liu F Yu M Zhang J Zhang Z Liu J Decreased regional activity and network homogeneity of the fronto-limbic network at rest in drug-naive major depressive disorder. Aust N Z J Psychiatry (2015) 49:550–6. 10.1177/0004867415577978 25788499

[27] Liu CH Li F Li SF Wang YJ Tie CL Wu HY Abnormal baseline brain activity in bipolar depression: a resting state functional magnetic resonance imaging study. Psychiatry Res (2012) 203:175–9. 10.1016/j.pscychresns.2012.02.007 23017873

[28] Guo W Cui X Liu F Chen J Xie G Wu R Increased anterior default-mode network homogeneity in first-episode, drug-naive major depressive disorder: a replication study. J Affect Disord (2018) 225:767–72. 10.1016/j.jad.2017.08.089 28938513

[29] Fox MD Raichle ME. Spontaneous fluctuations in brain activity observed with functional magnetic resonance imaging. Nat Rev Neurosci (2007) 8:700–11. 10.1038/nrn2201 17704812

[30] Zuo XN Di Martino A Kelly C Shehzad ZE Gee DG Klein DF The oscillating brain: complex and reliable. Neuroimage (2010) 49:1432–45. 10.1016/j.neuroimage.2009.09.037 19782143 PMC2856476

[31] Liu CH Ma X Wu X Fan TT Zhang Y Zhou FC Resting-state brain activity in major depressive disorder patients and their siblings. J Affect Disord (2013) 149:299–306. 10.1016/j.jad.2013.02.002 23474094

[32] Plante DT Jensen JE Schoerning L Winkelman JW. Reduced γ-aminobutyric acid in occipital and anterior cingulate cortices in primary insomnia: a link to major depressive disorder? Neuropsychopharmacology (2012) 37:1548–57. 10.1038/npp.2012.4 22318195 PMC3327859

[33] First M Spitzer R Gibbon M Williams J Structured Clinical Interview for DSM-IV Axis I Disorders. American Psychiatric Pub (1997).

[34] Hamilton M A rating scale for depression. J Neurol Neurosurg Psychiatry (1960) 23:56–62. 10.1136/jnnp.23.1.56 14399272 PMC495331

[35] Kennedy SH. Core symptoms of major depressive disorder: relevance to diagnosis and treatment. Dialogues Clin Neurosci (2008) 10:271–7. 18979940 10.31887/DCNS.2008.10.3/shkennedy PMC3181882

[36] Manber R Blasey C Arnow B Markowitz JC Thase ME Rush AJ Assessing insomnia severity in depression: comparison of depression rating scales and sleep diaries. J Psychiatr Res (2005) 39:481–8. 10.1016/j.jpsychires.2004.12.003 15992557

[37] Park SC Kim JM Jun TY Lee MS Kim JB Jeong SH Prevalence and clinical correlates of insomnia in depressive disorders: the CRESCEND study. Psychiatry Investig (2013) 10:373–81. 10.4306/pi.2013.10.4.373 24474986 PMC3902155

[38] Troxel WM Kupfer DJ Reynolds CF III Frank E Thase ME Miewald JM Insomnia and objectively measured sleep disturbances predict treatment outcome in depressed patients treated with psychotherapy or psychotherapy-pharmacotherapy combinations. J Clin Psychiatry (2012) 73:478–85. 10.4088/JCP.11m07184 22152403 PMC3310298

[39] Trivedi MH Bandelow B Demyttenaere K Papakostas GI Szamosi J Earley W Evaluation of the effects of extended release quetiapine fumarate monotherapy on sleep disturbance in patients with major depressive disorder: a pooled analysis of four randomized acute studies. Int J Neuropsychopharmacol (2013) 16:1733–44. 10.1017/S146114571300028X 23673347

[40] Lowe A Rajaratnam SM Hoy K Taffe J Fitzgerald PB. Can sleep disturbance in depression predict repetitive transcranial magnetic stimulation (rTMS) treatment response? Psychiatry Res (2013) 210:121–6. 10.1016/j.psychres.2013.04.028 23726870

[41] Chao-Gan Y Yu-Feng Z DPARSF. A MATLAB toolbox for “pipeline” data analysis of resting-state fMRI. Front Syst Neurosci (2010) 4:13 10.3389/fnsys.2010.00013 20577591 PMC2889691

[42] Jenkinson M Bannister P Brady M Smith S. Improved optimization for the robust and accurate linear registration and motion correction of brain images. Neuroimage (2002) 17:825–41. 10.1006/nimg.2002.1132 12377157

[43] Zang YF He Y Zhu CZ Cao QJ Sui MQ Liang M Altered baseline brain activity in children with ADHD revealed by resting-state functional MRI. Brain Dev (2007) 29:83–91. 10.1016/j.braindev.2006.07.002 16919409

[44] Stein MB Simmons AN Feinstein JS Paulus MP. Increased amygdala and insula activation during emotion processing in anxiety-prone subjects. Am J Psychiatry (2007) 164:318–27. 10.1176/ajp.2007.164.2.318 17267796

[45] Chand GB Dhamala M. The salience network dynamics in perceptual decision-making. Neuroimage (2016) 134:85–93. 10.1016/j.neuroimage.2016.04.018 27079535

[46] Bressler SL Menon V. Large-scale brain networks in cognition: emerging methods and principles. Trends Cogn Sci (2010) 14:277–90. 10.1016/j.tics.2010.04.004 20493761

[47] Uddin LQ. Salience processing and insular cortical function and dysfunction. Nat Rev Neurosci (2015) 16:55–61. 10.1038/nrn3857 25406711

[48] Touroutoglou A Bliss-Moreau E Zhang J Mantini D Vanduffel W Dickerson BC A ventral salience network in the macaque brain. Neuroimage (2016) 132:190–7. 10.1016/j.neuroimage.2016.02.029 26899785 PMC4851897

[49] Touroutoglou A Hollenbeck M Dickerson BC Feldman Barrett L. Dissociable large-scale networks anchored in the right anterior insula subserve affective experience and attention. Neuroimage (2012) 60:1947–58. 10.1016/j.neuroimage.2012.02.012 22361166 PMC3345941

[50] Deen B Pitskel NB Pelphrey KA. Three systems of insular functional connectivity identified with cluster analysis. Cereb Cortex (2011) 21:1498–506. 10.1093/cercor/bhq186 21097516 PMC3116731

[51] Cauda F Costa T Torta DM Sacco K D’Agata F Duca S Meta-analytic clustering of the insular cortex: characterizing the meta-analytic connectivity of the insula when involved in active tasks. Neuroimage (2012) 62:343–55. 10.1016/j.neuroimage.2012.04.012 22521480 PMC4782788

[52] Liu CH Jing B Ma X Xu PF Zhang Y Li F Voxel-based morphometry study of the insular cortex in female patients with current and remitted depression. Neuroscience (2014) 262:190–9. 10.1016/j.neuroscience.2013.12.058 24406440

[53] Liu J Ren L Womer FY Wang J Fan G Jiang W Alterations in amplitude of low frequency fluctuation in treatment-naïve major depressive disorder measured with resting-state fMRI. Hum Brain Mapp (2014) 35:4979–88. 10.1002/hbm.22526 24740815 PMC6869357

[54] Fitzgerald PB Laird AR Maller J Daskalakis ZJ. A meta-analytic study of changes in brain activation in depression. Hum Brain Mapp (2008) 29:683–95. 10.1002/hbm.20426 17598168 PMC2873772

[55] Halari R Simic M Pariante CM Papadopoulos A Cleare A Brammer M Reduced activation in lateral prefrontal cortex and anterior cingulate during attention and cognitive control functions in medication-naïve adolescents with depression compared to controls. J Child Psychol Psychiatry (2009) 50:307–16. 10.1111/j.1469-7610.2008.01972.x 19175815

[56] Wagner G Sinsel E Sobanski T Köhler S Marinou V Mentzel HJ Cortical inefficiency in patients with unipolar depression: an event-related FMRI study with the Stroop task. Biol Psychiatry (2006) 59:958–65. 10.1016/j.biopsych.2005.10.025 16458263

[57] Wang L Krishnan KR Steffens DC Potter GG Dolcos F McCarthy G. Depressive state- and disease-related alterations in neural responses to affective and executive challenges in geriatric depression. Am J Psychiatry (2008) 165:863–71. 10.1176/appi.ajp.2008.07101590 18450929

[58] Sunderajan P Gaynes BN Wisniewski SR Miyahara S Fava M Akingbala F Insomnia in patients with depression: a STAR*D report. CNS Spectr (2010) 15:394–404. 10.1017/S1092852900029266 20625372

[59] Adamo D Ruoppo E Leuci S Aria M Amato M Mignogna MD Sleep disturbances, anxiety and depression in patients with oral lichen planus: a case-control study. J Eur Acad Dermatol Venereol (2005) 29:291–7. 10.1111/jdv.12525 24754427

[60] Kok RM Reynolds CF III. Management of depression in older adults: a review. JAMA (2017) 317:2114–22. 10.1001/jama.2017.5706 28535241

[61] Smith SM Nichols TE. Threshold-free cluster enhancement: addressing problems of smoothing, threshold dependence and localisation in cluster inference. Neuroimage (2009) 44:83–98. 10.1016/j.neuroimage.2008.03.061 18501637

